# Pharmacotherapy of Primary Impulsive Aggression in Violent Criminal Offenders

**DOI:** 10.3389/fpsyg.2021.744061

**Published:** 2021-12-16

**Authors:** Alan R. Felthous, Bridget McCoy, Jose Bou Nassif, Rajat Duggirala, Ellen Kim, Fulvio Carabellese, Matthew S. Stanford

**Affiliations:** ^1^Department of Psychiatry and Behavioral Neuroscience, Saint Louis University School of Medicine, Saint Louis, MO, United States; ^2^Department of Medical Sciences, Surgery and Neurosciences, University of Siena, Siena, Italy; ^3^Hope and Healing Center and Institute, Houston, TX, United States

**Keywords:** primary impulsive aggression, pharmacotherapy, anti-impulsive aggression agents, criminal rehabilitation, conditional release, parole

## Abstract

Primary impulsive aggression (PIA) can be implicated as a common factor that results in an arrest, disciplinary, and restraint measures during confinement, and criminal recidivism after release. Evidence suggests that anti-impulsive aggression agents (AIAAs) can diminish or prevent impulsive aggression even when occurring with personality pathology such as borderline or antisocial personality disorder (ASPD), common conditions in offender populations. A previous review identified agents that have been subjected to controlled drug trials of sufficient quality, and subsequently, a decisional algorithm was developed for selecting an AIAA for individuals with IA. This selection process began with the five agents that showed efficacy in two or more quality studies from the earlier review. Today, 8 years after the quality review study, the present authors undertook this follow-up literature review. The aims of the present review were to survey the literature to identify and assess: (1) drug trials of comparable quality published since the 2013 review, including trials of the previously identified AIAAs as well as trials of agents not included in the earlier review; (2) severity of aggressive outbursts; (3) the materiality of risks or side-effects that are associated with individual AIAAs as well as antipsychotic agents commonly used to control clinical aggression; (4) efficacy of these agents in special populations (e.g., females); and (5) cost and convenience of each agent. Improved pharmacotherapy of PIA by addressing risks, side effects and practicality as well as the efficacy of AIAAs, should promote the rehabilitation and reintegration of some pathologically aggressive offenders back into the community.

## Introduction

### Primary Impulsive Aggression and the RNR Principles of Rehabilitation

The risk-need-responsivity (RNR; Andrews et al., 1990) principles have in recent years provided a conceptual model for the rehabilitation of criminal offenders (Wong and Gordon, 2013; Wong and Olver, 2015). Pharmacotherapy of primary impulsive aggression (PIA) fits well within this model. In the context to be addressed here, the risk would be unsuccessful post-release rehabilitation as measured by reduced relapse of a predisposing condition, rehospitalization, re-offending, re-imprisonment, or discontinuation of conditional release with resultant re-institutionalization. The RNR model typically includes psychotherapy, environmental adjustments, and social service programs to address the individual’s needs in order to reduce the specific risks that predispose to the general risk of failure of rehabilitation, in this case, aftercare. This approach also takes into account the individual’s responsivity or amenability to the proposed treatment.

Andrews and Bonta (2010) proposed the RNR model in 1990 as core to effective programming to assess and reduce recidivism. It has since been utilized in criminal justice systems in many countries, including parts of the United States. The core concepts behind the model are seemingly simple, direct intensive services to higher risk offenders, identify and target criminogenic needs, and provide treatment in a style that is responsive to the offender’s learning style and ability (Andrews and Bonta, 2010). However, the practical application of the model takes collaboration from criminal justice systems, social service systems, and behavioral health systems both in correctional and community settings, and bridging the gap from successful research models to daily practice has been a challenge. One of the criminogenic needs is antisocial personality traits or patterns that include impulsivity and aggression (Andrews and Bonta, 2010; Marlowe, 2018). The Substance Abuse and Mental Health Services Administration’s (SAMHSA) GAINS (Gather, Assess, Integrate, Network, and Stimulate) Center, in an effort to help professionals with the practical implication of the RNR model, has developed a Responsivity Assessment and Case Planning decisional tree (Marlowe, 2018). While pharmacotherapy for mental illness or impulsivity has not been identified at the core of the RNR model, treatment for impulsivity and aggression associated with antisocial personality patterns has been. Further studies looking at the role of pharmacologic treatment for PIA in correctional settings may add to the literature regarding the implementation of the RNR and other similar models targeting antisocial patterns.

While there has been a lack of studies examining the pharmacologic treatment of PIA, there have been studies examining the use of various therapies for violent offenders or impulsive aggression in correctional settings. In a 2019 meta-analysis, Papalia et al. (2019) examined whether psychological treatments for violent adult offenders were successful in reducing community recidivism. The psychological treatments included a variety of therapies and were required to have an aim to reduce violent, aggressive, or antisocial behavior. They found that, on average, those receiving therapy intervention had a significant reduction in violent recidivism by 10.2% points and in nonviolent recidivism by 11.2% points, suggesting that a variety of therapies may be effective in reducing both violent and nonviolent recidivism.

The two main categories of treatment programs for criminal offenders are general rehabilitation programs and programs that treat special offender populations such as those with severe mental illness, substance use disorders, intellectual disability, and sexual offending behavior (Felthous and Saß, 2006). Some offenders with PIA may benefit from therapeutic approaches such as the therapeutic community, token economy, and dialectic behavior therapy, but programs begun in prison are not typically continued after release and tend to not target impulsive aggression (Felthous and Saß, 2006).

Cognitive-behavioral therapy has been used to treat anger disorders (Deffenbacher, 2003; McCloskey, 2019) and within the context of residential criminal rehabilitation (Armelius and Andreassen, 2010). Other recent discussions have addressed psychotherapy of psychopathic and personality disorders in the context of criminal rehabilitation (e.g., Virdi and Trestman, 2015; Adshead and McGauley, 2021) and comprehensive outpatient treatment and management (Hill et al., 2021). To our knowledge, PIA and its treatment, including pharmacotherapy, have not been studied in the context of criminal rehabilitation during post-custodial release.

Here we focus on the pharmacotherapy of PIA, a condition that, left untreated, would pose a risk of aggressive behavior, which could result in failure at post-release reintegration into the community. While focusing in this article on pharmacotherapy, other interventions for this and related conditions should not be overlooked in designing an effective post-release rehabilitation and prevention of relapse/recidivism plan. Other treatment approaches which may be effective alone or in conjunction with pharmacotherapy include cognitive behavioral therapy (CBT is especially adaptable to the RNR model; Armelius and Andreassen, 2010; Gosse, 2013; Wong and Olver, 2015), dialectical behavioral therapy (DBT; Gallietta and Rosenfeld, 2012), and schema-focused therapy (SFT; Bernstein et al., 2007). The Reasoning and Rehabilitation (R&R) model has been used in both institutional and correctional settings (Ross et al., 1988) to reduce recidivism (Tong and Farrington, 2006). Consistent with the responsivity principle of the RNR model, the anti-impulsive aggression agents (AIAAs) must be efficacious in the individual, or, in other words, the individual must be responsive or amenable to the agent.

### Primary Impulsive Aggression and Personality Disorders

Impulsive aggression is one of the three types of aggression described in Barratt’s classification of aggression (Barratt, 1991; Barratt et al., 1997a,b; Barratt and Slaughter, 1998), the other two being medically related or secondary, and premeditated aggression, respectively. Barratt defined impulsive aggressive acts as aggressive acts with the absence of self-control which are easily triggered due to the individual’s sensitivity to provocation, after which they may express sadness or regret and a desire not to repeat the act (Barratt, 1991). This is a Strombolian emotionally charged overreaction culminating in an aggressive act during which information processing appears insufficient. Impulsive aggression is comparable to the “reactive” aggression of Dodge (Dodge, 1991; Dodge and Coie, 1998) and the effective aggression of Raine (Raine et al., 1998). Impulsive aggression is essentially the mirror opposite of premeditated aggression, which is controlled, planned, not emotionally driven, and self-serving (Felthous and Barratt, 2003; Felthous and Stanford, 2021).

Aggression secondary to another known mental disorder may be impulsive or premeditated. It typically subsides when the primary mental disorder of which the aggression is symptomatic is effectively treated. For example, if the aggression is part of mania in bipolar disorder, the aggression typically responds to appropriate pharmacotherapy of the bipolar disorder. The aggression considered here has more recently been qualified with “primary” to more unequivocally distinguish it from impulsive aggression that is secondary or medically related.

Antisocial personality disorder (ASPD) was formulated by polythetic behavioral criteria derived from Robins (1979) research. This disorder begins in childhood with features of conduct disorder with the pattern of the rule violation and antisocial behavior extending into adulthood. Today’s DSM criteria (American Psychiatric Association, 1980; American Psychiatric Association, 2013) are essentially the same as those in the third edition published in 1980 and include getting into fights which could represent impulsive or premeditated aggression.

Impulsive aggression can also be one of the polythetic diagnostic criteria of borderline personality disorder (BPD) in the DSM-5. The core feature of this personality disorder is instability, instability of emotions, relationships, and one’s identity and sense of direction in life.

Primary impulsive aggression can, occur in the context of antisocial, borderline, or other personality disorder, despite the personality disorder relative exclusionary criteria of the DSM-5 for the intermittent explosive disorder (IED; American Psychiatric Association, 2013), the DSM-5 diagnostic equivalent of primary impulsive disorder (Felthous and Stanford, 2021). Within the DSM, but only since the Fifth Edition in 2013, the IED can be one of two types: In Type 1 the angry outbursts are frequent but mild in intensity, whereas in Type 2 they are infrequent but intense, two types which could inform the selection of an AIAA (Felthous and Stanford, 2015).

### Impulsive Aggression and the PCL-R Factors and Facets

Impulsive aggression can also be a non-pathognomonic feature of PCL-R psychopathy, i.e., psychopathy, as measured by the Psychopathy Checklist-Revised (PCL-R), developed by Hare (1991, 2003, 2021). Similar to but also in contrast to ASPD, psychopathy is conceptualized more like a constellation of traits than antisocial behaviors, the constellation of traits that are interpersonal, affective, and of lifestyle, but also behaviors that include deception, manipulation, irresponsibility, impulsivity and stimulation-seeking. Behavior can be unethical and antisocial, but not always criminal. Individuals with psychopathy show deficient capacity to experience empathy, guilt or remorse and poor behavioral control (Hare, 1999, 2021).

One would expect Factor 2, which includes criminal behavior and impulsive aggression, would be more predictive of criminal recidivism, an oft-repeated theme in the risk assessment literature. Yet Factor 1 itself is strongly predictive of violence (Olver et al., 2013) and offenders’ dropping out of treatment (Olver and Wong, 2011), outcomes that would favor recidivism and the need for rehabilitative attention.

The PCL-R is divided into two factors or clusters of features. Factor 1 corresponds to the interpersonal and affective aspects, whereas Factor 2 represents the socially deviant lifestyle. Studies are not consistent with regard to which of the two factors is more predictive of violence. The PCL-R (Hare, 1991, 2003) provides a widely recognized and reliable measure of psychopathy (Hare, 2021). It is as predictive of general and violent offending as instruments developed only to predict such outcomes (Hart et al., 1988; Hare, 2021). According to Hare, Factor 1 is counterintuitively more predictive of violence than Factor 2 (Hare, 2021). In contrast, in a recent study of 445 Korean men incarcerated then released, Facet 2 showed the strongest effect size in predicting violent recidivism (Sohn et al., 2019). Other studies found behavioral characteristics of psychopathy were more predictive of both general and violent recidivism (Walters et al., 2008; Kennealy et al., 2010). A study in Pakistan using Levenson’s Self Report Psychopathy Scale (Levenson et al., 1995) found egocentricity and antisocial behavior to be predictive of violent offending (Shagufta, 2020). A Finish study assessed 40 violent female offenders with the PCL-R and then followed for 8 years after release from prison or psychiatric hospital. The authors concluded that impulsivity was statistically predictive of violent reoffending, whereas the main PCL-R scores were not (Weizmann-Henelius et al., 2015).

Azevedo et al. (2020) in Portugal distinguished premeditated from impulsive aggression by using an instrument to make this distinction in 134 male prisoners. They administered the PCL-R, the Addiction Severity Index European version, the Barratt Impulsivity Scale (BIS-11), and importantly, the Impulsive Premeditated Aggression Scale. Of 96 offenders diagnosed with ASPD, 71.9% had impulsive aggression and 28.1% premeditated aggression. Both groups were similar with regard to substance use disorder and BIS-11 scores. Compared with subjects with premeditated aggression, those with impulsive aggression had lower Factor 1 scores (*p* < 0.01) and facet 1 (interpersonal) scores (*p* < 0.05). There was an inverse relationship between psychopathy and impulsive aggression, the latter showing a stronger relationship with ASPD.

In any event, PIA can be regarded either as a separate but potentially co-occurring disorder with ASPD, BPD, or PCL-R psychopathy, somewhat analogous to a substance use disorder or as a domain of the personality disorder or psychopathic condition (Felthous and Stanford, 2021), a co-occurring disorder or domain which may be amenable to therapeutic approaches including pharmacotherapy. Indeed, research demonstrating efficacy was conducted on personality disordered populations well represented with ASPD and BPD.

### Primary Impulsive Aggression and Post-release

Primary impulsive aggression can be implicated as a common factor in behavior that results in an arrest, disciplinary and restraint measures after confinement, and criminal recidivism after release. Yet studies examining the relationship between impulsive aggression with criminality and criminal recidivism are lacking. Impulsive aggression is a diagnostic criterion for ASPD and BPD (American Psychiatric Association, 2013), two conditions commonly diagnosed in correctional populations (Teplin, 1994; Conn et al., 2010). A systematic review of 23,000 prisoners from 12 countries found that 47 percent of male prisoners and 21 percent of female prisoners had antisocial personalities (Fazel and Danesh, 2002). Virdi and Trestman (2015) report the ASPD prevalence in correctional settings varies from 35 to 75%. Jail inmates with ASPD are charged with more offenses and more violent offenses than controls without ASPD (Riser and Kosson, 2013). Multiple studies have shown that in prison populations, 35–50 percent of prisoners have BPD, with the percentages consistently higher among female prisoners (Black et al., 2007; Virdi and Trestman, 2015). ASPD and BPD are also over-represented in jail populations (Teplin, 1994; Drapalski et al., 2009; Conn et al., 2010).

Antisocial personality disorder (Ogloff et al., 2015) and elevated psychopathy are consistently associated with criminal recidivism (Kroner and Yessine, 2013) after release from prison.

A recent meta-analysis did not support a direct link between ASPD, impulsive aggression and criminality (Farrington et al., 2017); whereas Ogloff et al. (2015) have found that co-occurring ASPD, where the co-occurring disorder is psychotic, affective, or substance use disorder, is correlated with more serious and frequent offending. In general, studies report the predictive value of psychopathy, ASPD, and BPD, disorders known for impulsive aggression (DSM-5; American Psychiatric Association, 2013). But with few exceptions, studies following release from prison or forensic security hospitals have not specifically examined impulsive aggression (Vitacco et al., 2014), a dynamic condition that could respond to appropriate treatment.

### Pharmacotherapy of Primary Impulsive Aggression

Studies have suggested that specific medicines, already FDA approved for the treatment of other conditions, can reduce or prevent aggressive episodes of PIA, even within the pathological context of a personality disorder, such as borderline or ASPD (Barratt et al., 1997a,b; Lee et al., 2008, 2019b). Although not formally tested for PCL-R assessed psychopathy (Hare, 1991, 2021) in these studies, there is reason to expect that efficacy should exist even within the context of psychopathy (Felthous, 2016; Felthous et al., 2018; Felthous and Stanford, 2021).

Regrettably, many studies on the pharmacotherapy of aggression did not define and diagnose PIA (Barratt and Slaughter, 1998; Felthous and Stanford, 2015). The Cochrane review on pharmacological interventions for ASPD, for example, used aggression as an outcome measure, but this was a study of ASPD, not impulsive aggression (Khalifa et al., 2010). This has likely accounted for disparate results.

Another flaw in earlier reports on the effect of specific agents on impulsive aggression or IED was that the subjects were diagnosed with more than one major Axis I disorder. In some reports, they were simultaneously treated with other psychotropic agents for the other disorders(s).

Aggression secondary to another mental disorder can also be impulsive in nature. Therefore, the aggression considered here is sometimes distinguished by the qualifier “primary.”

The value of selecting well-tolerated, practical-to-administer, and efficacious AIAAs for individuals with PIA to the criminal justice system should be self-evident. Effective control of violent intermittent aggression can prevent some crimes and rehabilitate and prevent recidivism where criminal behavior is due to PIA. Yet, the extensive body of research on release planning and aftercare of imprisoned offenders singularly lacks consideration of PIA and its pharmacotherapeutic treatment.

Compared to PIA, much more attention has been directed at treatment of substance use disorders to improve criminal rehabilitation and reduce recidivism (Hollenshead et al., 2021; Kiefer and Frischknecht, 2021). Substance use is appropriately addressed in programs supporting transitions of prisoners into the community (Meyer and Alice, 2015), whereas impulsive aggression, in contrast, is not typically a condition of focused rehabilitative treatment. Offenders with substance use disorders show increased employment and reduced substance use if subjected to longer lengths of stay in therapeutic communities or recovery homes after release from jail or prison (Jason et al., 2015). Aftercare relapse prevention programs have reduced criminal recidivism for female offenders with substance problems by a factor of 10 (Matheson et al., 2011). A recent study showed higher rates of recidivism among released inmates with only a substance use disorder than those with both substance use disorder and mental illness (Zgoba et al., 2020).

The purpose of this perspective review is to provide information that can usefully guide clinicians in selecting the most appropriate AIAA in treating PIA, thereby supporting the RNR model in reducing criminal recidivism and promoting post-release successful reintegration of offenders into the community. Foremost, therefore, was the need to assess the current state of knowledge regarding the efficacy of AIAAs. Accordingly, an important aim of this review was to identify and assess drug trials in the treatment of PIA published since the 2013 review, studies of the same quality as determined by the same measures. This was accomplished using the same method as that of the earlier review. Secondly, of those drugs with sufficient evidence of efficacy, to update the specific risks and side effects that are so material as to inform AIAA selection in treating individual patients with PIA. Also relevant to AIAA selection are severity and frequency of aggressive outbursts, the relative efficacy of each AIAA based upon the quality drug trials, and the agent’s cost and convenience of administration. And finally, we identify critical areas of research for developing more efficacious AIAAs and effective strategies for the pharmacotherapy of PIA.

## Updated Review of Quality AIAA Trials

### Selection of Drug Trial Studies

Using MEDLINE and PsycINFO, the authors attempted to identify all controlled studies in the English language, published since 2013, that tested for drug efficacy in the pharmacotherapy of PIA. As with the previous article published in 2013, the present search did not include studies in the pharmacotherapy of acute agitation on an emergency basis, but rather the ongoing administration of a medication to prevent or reduce the frequency and intensity of future acts of aggression. The present search aimed to identify new agents with potential efficacy in the treatment of impulsive aggression, and uncover additional evidence pertaining to the efficacy of previously identified agents. Particular interest was given to the antipsychotic agents, recognizing their common use in the management of impulsive aggression in inpatient and correctional settings.

Between January and March 2021, an exhaustive search of the two databases mentioned above was performed, with different variations of the search terms “pharmacotherapy” and “impulsive aggression” being used in multiple combinations (see Table 1). An expanded search was performed using terms related to “antisocial personality,” “borderline personality,” as well as searches including specific medications that have been hypothesized to play a role in the management of impulsive aggression (see Table 2). A repeat search using MeSH terms was used in both databases.

**Table 1:** List of terms searched for in the initial literature review.

Pharmacotherapy Psychopharmacology Pharmacology Treatment Drug therapy (MeSH) Psychopharmacology (MeSH)	Aggression Impulsive aggression Impulsive/impulsiveness/impulsivity Intermittent explosive disorder Antisocial personality (disorder) Borderline personality (disorder) Personality disorder Aggression (MeSH) Disruptive, impulse control, and conduct disorders (MeSH) Personality disorders (MeSH)

Combinations of all the terms from both columns were used in both databases, with MeSH terms only pertaining to the Pubmed.org database.

**Table 2:** List of pharmaceutical agents searched for in the second literature review.

Antipsychotics	Mood stabilizers/Antiepileptics	Others
Quetiapine Olanzapine Haloperidol Risperidone Clozapine Aripiprazole Ziprasidone	Lithium Lamotrigine Valproate Carbamazepine Oxcarbazepine Phenytoin	Fluoxetine Sertraline Escitalopram SSRI Propranolol Pindolol Beta blockers Clonidine Antihypertensives (MeSH)

Combinations of these agents with the terms from the right-sided column in Table 1 were used in both databases, with MeSH terms only pertaining to the Pubmed.org database.

### Criteria of Inclusion and Exclusion

To be included in this search, the study must have had at least one comparison group to control for the placebo effect. The study must have adequately defined and diagnosed the presence of impulsive aggression by approximating the definition of impulsive aggression provided by Barratt (1991) and Barratt et al. (1997b), applying the criteria for IED from one of the DSMs since the third edition, or a reasoned modification of such criteria. Studies that included other primary disorders such as epilepsy, intellectual disability, autism spectrum disorder, attention-deficit hyperactivity disorder, substance use disorders, or other psychotic and affective disorders were excluded. Studies focusing exclusively on children and adolescents were also excluded. Studies that focused primarily on personality disorders, specifically antisocial and BPDs, were included, regardless of their co-occurrence with the IED.

Any drug trials that met these inclusion and exclusion criteria were then further assessed using the same quality checklist used in the 2013 review. By this method, we hoped to identify studies of the same quality published since 2013.

### Results of Search for AIAA: Drug Trial Studies

Literature searches for studies assessing drug efficacy in the pharmacotherapy of aggression found seven peer-reviewed publications. Where the aggressive population studied did not carry an exclusionary diagnosis listed above. Review of those studies found that none met the criteria for inclusion in the quality comparison. The primary reason studies were not included was that they did not specifically assess impulsive aggression but evaluated aggression in general (see Table 3).

**Table 3:** Reasons studies were excluded from quality comparison.

Reason	*n*	% of total
Not specifically impulsive aggression	5^*^	62.5
Not specifically treatment	1	12.5
Meta-analysis	1	12.5
Other	1	12.5

* Of the eight studies that were considered in our analysis, five were excluded since they did not specifically measure impulsive aggression. These include a 2017 study on melatonin and aggression (Liu et al., 2017), a 2015 study on the role of fluoxetine on inflammatory markers in IED subjects (Coccaro et al., 2015), a 2013 study on the role of omega-3 supplementation of valproic acid in borderline personality disorder (BPD), and two studies pertaining to SSRIs and irritability (Jha et al., 2020; Towbin et al., 2020).

## Efficacy of AIAAs

The previous review (Felthous et al., 2013) confirmed the efficacies of three anticonvulsants: carbamazepine, phenytoin, and valproate for impulsive aggression (Barratt et al., 1997a,b; Hollander et al., 2005; Stanford et al., 2005). The mean serum levels for the anticonvulsants were below or on the lower end of the therapeutic ranges for seizures: phenytoin 3.3 μg/ml (10–20 μg/ml), carbamazepine 4.3 μg/ml (4–12 μg/ml), and valproate 39.2 μg/ml (50–120 μg/ml; Stanford et al., 2005).

Phenytoin, in particular, significantly reduced aggressive acts by 71% and the intensity of aggression by 60% (Barratt et al., 1997a). It also had positive effects on information processing as measured by event-related potentials (ERPs). In the Barratt study, the reduced amplitudes of P3 ERP waveforms among impulsive aggressive subjects were normalized during the phenytoin condition but not during the placebo condition (Barratt et al., 1997a; Given phenytoin’s efficacy in reducing impulsive aggression below the therapeutic range, females with reliable forms of contraception potentially could be studied in the future).

Valproate was found to be effective in patients with impulsive aggression and co-occurring BPD (Hollander et al., 2003, 2005). The treatment differences favoring valproate were found early in this study, suggesting that its effects on irritability may contribute to its treatment effect on impulsive aggression (The efficacy of valproate in BPD may be attributed to patients with BPD having a greater lifetime history of aggression). Additionally, patients with cluster B personality disorders may share neurobiological features with patients with bipolar spectrum disorders, who respond to valproate.

Not all AEDs have shown efficacy in the treatment of PIA. Levetiracetam, in a study of males and female showed no evidence of efficacy in treating impulsive aggression (Mattes, 2008). In another study with levetiracetam of males and females, there was no evidence of efficacy in patients with impulsive aggression (*n* = 40; Mattes, 2009).

Central serotonergic dysfunction is associated with impulsive aggression (Fanning et al., 2021). Coccaro et al. (2009) found that fluoxetine was associated with full remission of aggressive behaviors in 29% of patients and full or partial remission in 46% of patients with IED, which was defined by the A criteria of IED. Although these results are encouraging, fluoxetine should not be considered a “magic bullet.” Of note, data suggests at the start of treatment, there may be an increase in impulsive aggressive behavior so this should be taken into account as these treatments may ultimately be efficacious in reducing PIA behavior.

Limitations in comparing the efficacies of the AIAAs included different measurements of success and statistical analyses. Additionally, longer trials would better elucidate the efficacy of AIAAs. For example, phenytoin, which does not have an FDA-approved use for psychiatric disorders, has been shown to be effective in treating impulsive aggression. A 6-week trial of phenytoin had a 71% decrease in the frequency of aggressive acts (Barratt et al., 1997a). Longer trials of 16 weeks had lower frequencies, 41 and 57% decreases, suggesting that phenytoin as an impulsive aggressive agent may lose its efficacy at least for some overtime (Stanford et al., 2001; Houston and Stanford, 2006). Carbamazepine, which has FDA-approved psychiatric use for mania, had a slower onset of efficacy than phenytoin and valproate in an 8-week trial (Stanford et al., 2005). The onset of treatment would have important implications when tailoring treatment for individuals (Stanford et al., 2005). These findings suggest that antiepileptics may have varying efficacy over time, with phenytoin potentially losing effectiveness and carbamazepine needing more time for its effects.

The next steps to strengthen the efficacy of these agents for eventual FDA approval for impulsive aggression/IED include studying different populations of IA patients, investigating further non-antiepileptic agents, and designing longer trials. Additionally, studies should focus on factors and specific patient characteristics that are associated with favorable responses to the agents, particularly demographic factors such as gender.

## Severity and Frequency of Outbursts

Proper assessment includes a description of the angry outbursts as well as their severity and frequency, following the DSM criteria for the IED (American Psychiatric Association, 2013). According to the DSM-5, IED can be more specifically diagnosed as Type 1 and Type 2, with Type 1 designating recurrent, impulsive aggression that is frequent but less severe, whereas Type 2 represents severe but less frequent outbursts (American Psychiatric Association, 2013). This diagnostic dichotoimization does not exclude the possibility that impulsive aggressive outbursts can be both frequent and severe.

Diagnosing impulsive aggression as Type 1 and 2 may inform the selection of the most appropriate AIAA. For Type 1, fluoxetine may be the most reasonable first choice, not only for its convenience of administration and relatively favorable side-effect profile but because research suggests its efficacy with Type 1 IED (Coccaro and Kavoussi, 1997; Lee et al., 2008, 2019b). The more severe outbursts manifested by assault and property destruction of Type 2 may, in contrast, warrant starting with lithium or an anticonvulsant AIAA (Felthous and Stanford, 2015). Research demonstrating the efficacy of phenytoin in controlling impulsive aggression was conducted on prisoners who manifested the severe form of impulsive aggression corresponding to Type 2 (Barratt et al., 1997a,b). Initially recommended by Felthous and Stanford (2015), this dichotomous method of AIAA selection based upon Type 1 and 2 IED determination was subsequently endorsed by Lee et al., (2019b) but with the caveat that females of childbearing age be tried on fluoxetine before starting an anticonvulsant. If however, the aggression is serious and dangerous, lithium may be a reasonable selection for such women as it has been shown to mitigate severe aggression, at least in male subjects, and now after decades of experience with lithium in pregnancy, it is considered to present a lower risk to pregnancy than earlier thought (Cohen et al., 1994; Felthous, 2013).

## Materiality of Risks or Side Effects

In this section, we focus on the individual agents with consideration of their risks and side effects and ease of administration. Beyond evidence-based efficacy, the drug’s side effect profile and risk of specific side effects inherent in the patient should be taken into consideration when choosing an agent. This is especially important where AIAAs are used for an indication not approved by the FDA. Most of the literature available about the side effects of these agents that are antiepileptic comes from studies in which doses were effective in the treatment of a seizure or other mental disorder. Some of the side effects registered may not be as prevalent at the lower doses used to treat PIA. However, not all side effects are dose-dependent, as we attempt to delineate in the discussion below.

When determining the most appropriate agent for an individual, it is important to consider additional medications that the individual is already taking and any potential interactions between drugs. This can mean adjusting doses, discontinuing the other drug, or selecting an alternative AIAA.

### Phenytoin

Phenytoin is an antiepileptic agent well known and used by neurologists. Unlike many of the other agents discussed, phenytoin does not have an approved psychiatric use, however, it has been shown in some studies to be effective in treating impulsive aggression.

Common side effects include dizziness, lethargy, skin hypersensitivities, coarsening of facies, hirsutism, and neurotoxicity (dizziness, lethargy, nystagmus, and unsteady gait; Trescher and Lesser, 2008). A systematic review and meta-analysis comparing levetiracetam (inefficacious for AIAA) and phenytoin showed that rash, anaphylaxis, arrhythmia, and hyponatremia were more common in those taking phenytoin (Lee et al., 2019a). The side effects of phenytoin are primarily dose-related (Trescher and Lesser, 2008), and cardiac arrhythmias are seen most commonly when using intravenous phenytoin (De Toledo and Ramsay, 2000). Notably, phenytoin seems to be effective in PIA at serum levels of 4–5 mg/ml, which is substantially lower than the range usually required for treatment of seizures, 10–20 mg/ml (Barratt et al., 1997a). Most of the side effects from phenytoin that have been reported come from studies utilizing doses high enough to target seizures, further, many of the side effects of phenytoin are doserelated, so it may be well tolerated at doses needed for treatment of PIA.

Phenytoin impairs vitamin D absorption and causes hypocalcemia in non-ambulatory patients (Trescher and Lesser, 2008). Because of this risk, caution should be taken in prescribing phenytoin to those who are non-ambulatory, elderly, and postmenopausal women. Phenytoin is also noted to interfere with folate metabolism and can cause megaloblastic anemia (Trescher and Lesser, 2008).

The use of phenytoin in women of childbearing age should be carefully considered as it may have several different impacts on reproduction. It may impact the metabolism of oral contraceptives and decrease their efficacy (Trescher and Lesser, 2008), so alternative agents may be advised, and if phenytoin is chosen, alternative birth control methods should be pursued. Additionally, because of phenytoin’s impact on folate metabolism, folate supplementation is recommended for any pregnant women or women trying to conceive taking phenytoin. Finally, phenytoin has been associated with a pattern of congenital malformations called fetal hydantoin syndrome. The features of this syndrome include pre-natal and post-natal growth deficiency, developmental delay and microcephaly, along with distal pharyngeal hypoplasia and dysmorphic craniofacial abnormalities (Eroglu et al., 2008).

Phenytoin, along with most of the older antiepileptic drugs, is an enzyme-inducing agent which leads to many of its clinically significant interactions with other drugs. This mechanism causes it to stimulate the metabolism and decrease the concentration of many medications, including valproate, lamotrigine, carbamazepine, oxcarbazepine, topiramate, and many benzodiazepines (Perucca, 2006). Phenytoin levels can be increased if administered with medications that inhibit metabolizing enzymes. There are a number of potentially interfering drugs that may lead to increased levels of phenytoin in a variety of classes, including, but not limited to, antiepileptics (oxcarbazepine), antidepressants (fluoxetine, fluvoxamine, sertraline, and trazodone), antimicrobials, antineoplastics, and a number of miscellaneous medications (Perucca, 2006). Similarly, there are a number of medications in a variety of classes that may be decreased by co-administration with phenytoin due to its enzyme-inducing properties these interactions are similar to those seen with carbamazepine.

### Carbamazepine and Oxcarbazepine

Carbamazepine is a mood stabilizer and antiepileptic agent used by psychiatrists and neurologists. While not FDA approved for the treatment of PIA, two studies have shown it to be effective (Felthous et al., 2013). Oxcarbazepine, also a mood stabilizer and antiepileptic, has been studied in the treatment of PIA (Mattes, 2005, See also Bellino et al., 2005). However, as the two medications have similar mechanisms of action and oxcarbazepine is generally better tolerated, they are both reviewed here.

Oxcarbazepine and carbamazepine share structural and mechanistic characteristics, and their mechanisms of action are thought to be similar, however, oxcarbazepine seems to be better tolerated than carbamazepine. Neurotoxicity, including sedation, ataxia, diplopia, and nystagmus, is fairly common during treatment with carbamazepine, particularly at higher doses and early in the course of treatment (Wang et al., 2017). However, the dose of carbamazepine needed to target impulsive aggression is thought to be around 450 mg daily, lower than the typical dose needed to treat manic symptoms (Jones et al., 2011), suggesting that neurotoxicity may not be as problematic during treatment of PIA. Carbamazepine commonly causes benign hematological, dermatological, electrolyte, and hepatic problems (Wang et al., 2017). Consequently, baseline labs, including a CBC with differential, platelets and hepatic functioning, should be obtained, and periodically monitored. As carbamazepine can cause a benign or serious rash, it is a side effect that requires close monitoring. The rate of rash development has been reported to be somewhere between 2 (cited by the manufacturer) and 16%, but the rate of severe rash incidence is estimated to be somewhere between 0.5 and 1% (Kramlinger et al., 1994). Similarly, it is estimated that the incidence of leukopenia is between 2 and 14%, with the majority of those cases being benign, and the incidence of aplastic anemia is estimated to be around 1 in 125,000 people (Tohen et al., 1995). There is a noted increased risk of serious dermatological effects such as toxic epidermal necrolysis and Stevens-Johnson syndrome in certain Asian populations who carry the HLA-B*1502 allele. Due to this increased risk, carbamazepine carries a black box warning, and the US prescribing information recommends screening patients with ancestry in genetically at-risk populations (Asian) for the allele prior to initiating treatment. Carbamazepine is also a known teratogen. A review of all published cohort studies with the goal of identifying its teratogenic effects showed an overall incidence for a major congenital malformation of 3.3%, and the only specific major congenital malformation associated with carbamazepine was spina bifida (Jentink et al., 2010).

Oxcarbazepine has been noted to be more tolerable, does not carry the same black box warning, and is not a known teratogen. It has a lower propensity to cause rash and neurotoxicity (Wang et al., 2017). Oxcarbazepine may cause elevations in transaminases and gastrointestinal upset. Notably, hyponatremia is more common in oxcarbazepine than in carbamazepine. This is typically asymptomatic but may not be, particularly in those already at risk for electrolyte abnormalities, such as the elderly (Wang et al., 2017). While oxcarbazepine does not carry the black box warning regarding serious dermatologic reactions and the HLA-B*1502 allele, the prescribing information states that screening for the HLA-B*1502 allele should be considered genetically at-risk populations. Oxcarbazepine has not been associated with congenital malformations thus far in humans, however, it can reduce levels of oral contraceptives by up to 50%, which should be considered when prescribing to women of childbearing age (Pratoomsri et al., 2006).

Carbamazepine is an enzyme-inducing agent and has many interactions with other drugs. The depth of those interactions is beyond the scope of this article, however, we will briefly discuss some that may be particularly relevant. Many medications may be decreased if given with carbamazepine, including carbamazepine itself which is an autoinducer. It may also cause decreased concentration of multiple antidepressants (bupropion, citalopram, mirtazapine, sertraline, and tricyclics), antipsychotics (aripiprazole, clozapine, haloperidol, olanzapine, and risperidone), some benzodiazepines, buprenorphine, warfarin and notably many other anticonvulsants, some of which are also used for PIA or other psychiatric illnesses (lamotrigine, oxcarbazepine, phenytoin, and valproate; Wang et al., 2017). Carbamazepine also decreases the serum concentration of a number of medications used for primary medical illnesses, including some anti-infectives, analgesics, steroids, muscle relaxants, and immunosuppressants. Additionally, there are a number of medications that increase the concentration of carbamazepine. These include some antibiotics, antifungals, and calcium channel blockers. Notably, some antidepressants increase the concentration of carbamazepine, including fluoxetine (Wang et al., 2017). Oxcarbazepine is a moderate enzyme inducer and therefore has fewer and less robust drug–drug interactions compared to carbamazepine.

### Valproate

Valproate is commonly used in the treatment of bipolar disorder and epilepsy and, while not FDA approved, has been shown to be effective in managing PIA. Both tremor and gastrointestinal side effects commonly occur with valproate and are dose-related and benign. There may be the benign elevation of hepatic transaminases, however, no longer-term studies in the past approximately two decades that followed individuals treated with valproate have shown hepatic dysfunction or significant worsening of baseline hepatic function (Bowden, 2017). Leukopenia and thrombocytopenia may occur and are typically mild (Bowden, 2017). Due to the potential impact on blood cells, platelets, and hepatic function, baseline and periodic monitoring of CBC with differential, hepatic function, and serum drug levels are recommended. Weight gain may be a significant and limiting side effect. An increase of 3–24 pounds has been seen in 3–20% of those taking valproate (Bowden, 2003). A cross-sectional study found women who had reported taking valproate previously had higher rates of the polycystic ovarian syndrome (PCOS; Joffe et al., 2006). The correlation between valproate and PCOS remains unclear, but obesity is likely a contributing factor and should be considered when prescribing valproate to an already obese or overweight female. Valproate is a known teratogenic agent. It has been associated with an increased incidence of birth defects, including neural tube defects, in 1–4% of infants exposed to valproate in the first 10 weeks of gestation (Bowden, 2017). Because of this elevated risk, an alternative agent should be used in pregnant women, especially during the first trimester and women trying to conceive. Caution is also advised, and other agents should be considered for women of childbearing age who are sexually active and not consistently using a reliable method of birth control.

While many older anticonvulsants induce liver enzymes that metabolize many medications, valproate is an enzyme inhibitor. Additionally, it is highly bound to plasma proteins. Both of these features lead to some of its interactions with other drugs. Two of the most clinically important interactions caused by valproate’s inhibition of liver enzymes are interactions with lamotrigine and phenobarbital. The inhibition of enzymes leads to decreased metabolism and increased levels of both lamotrigine and phenobarbital (Perucca, 2006). Another clinically significant interaction occurs with aspirin. Both valproate and aspirin are highly bound to plasma proteins and mutually displace each other, however, aspirin also inhibits beta-oxidation, which is partially responsible for valproate’s metabolism. This combination can lead to high free valproate concentrations with clinical symptoms and toxicity, while total valproate levels may only be mildly increased (Sandson et al., 2006). These interactions should be considered when prescribing valproate, and the interaction with aspirin should be discussed with the patient as this is a commonly used over-the-counter medication.

### Lithium

Lithium has possibly been underutilized, at least in the United States, for treatment of bipolar disorder due to concerns over its side effect profile (Post, 2018). It was among the agents previously identified as efficacious in treating PIA and may be considered as one of the first agents to use, particularly for someone with Type 2 PIA. The two main side effects that have been noted to decrease compliance are cognitive impairment and weight gain (Bowden, 1998). Cognitive impairment is typically expressed as difficulty with short-term memory and information processing (Bowden, 1998). Some weight gain with lithium is common with reports of 20–77% of lithium-treated patients experiencing weight gain, and in some, the weight gain is clinically significant, greater than 7% of baseline weight (McKnight et al., 2012). Gastrointestinal side effects, including nausea and diarrhea, are common with lithium use but do not seem to be a common cause for discontinuation of treatment (Gitlin, 2016). A benign postural tremor of the hands is seen frequently with lithium use and should be distinguished from the coarser, more severe, and widespread (affecting parts of the body outside of the hands) tremor that is associated with lithium toxicity (Gitlin, 2016). Lithium has been associated with some cardiac changes, including bradycardia, sinus node dysfunction and T-wave changes on an electrocardiogram (Kamali et al., 2017).

Lithium is known to impact three distinct organ systems, especially with use over a prolonged period of time: the thyroid gland, parathyroid gland, and kidneys. Studies suggest that those taking lithium have a lower glomerular filtration rate (GFR) than matched controls (McKnight et al., 2012), however, the lower GFR is not correlated with time on lithium, nor does it necessarily progress with treatment (Gitlin, 2016). There is a sub-group that has a progressive lowering of GFR and increase in creatinine with continued lithium use (Gitlin, 2016). The data on the risk of progression to chronic kidney disease is scarce and at times conflicting, largely due to varied definitions of renal insufficiency. However, the risk of advancement to end-stage renal disease is considered relatively low, there may be an increased risk compared to healthy control, but it has been suggested to be as low as 0.5% (McKnight et al., 2012). Lithium has been associated with thyroid impairment at rates reported between 5 and 35% (American Psychiatric Association, 2002). Some of the discrepancies in reported prevalence is explained by various outcomes being measured. Associations with abnormal laboratory values, goiter development, subclinical hypothyroidism, and hypothyroidism, are all examples of definitions of thyroid impairment used for studies (Gitlin, 2016). Lithium is associated with an increase in calcium and parathyroid hormone (PTH) levels of 10% (McKnight et al., 2012). This elevation can lead to clinical signs of hypercalcemia such as renal stones, osteoporosis, and weakness (Gitlin, 2016).

Lithium has a narrow therapeutic index, and patients are at risk of becoming toxic at levels above the index. Toxicity can typically be avoided by monitoring drug levels periodically. Additionally, both the prescriber and the patient should be aware that changes in fluid intake, gastrointestinal sickness, or changes in certain medications may impact the lithium level. Symptoms of lithium toxicity range from weakness, ataxia and poor attention to slurred speech, gross tremor, and confusion and at extremely high lithium levels, patients may require emergent attention and dialysis (Gitlin, 2016).

Lithium has historically been associated with an increased risk of congenital malformations, specifically Ebstein’s anomaly when taken during early pregnancy. However, more recent data suggests that the teratogenic risk of lithium exposure during pregnancy is much lower than previously thought (Cohen et al., 1994). The prevalence of this anomaly in first-trimester lithium exposed pregnancies is higher than the general population, with a relative risk of 10–20 times the risk in the general population. However, the anomaly is rare even in the general population, so the absolute risk of Ebstein’s anomaly in lithium exposed pregnancies is estimated to be between 0.05 and 0.1% (Cohen and Rosenbaum, 1998). While the absolute risk of teratogenicity is relatively low in lithium exposed pregnancies, women should still be engaged in reproductive risk counseling if already taking lithium or considering the use of lithium during pregnancy (Cohen and Rosenbaum, 1998).

As lithium may impact multiple organ systems with use, it is recommended to get baseline labs to determine kidney function (at least BUN and creatinine), thyroid function (at least TSH), a pregnancy test if indicated, and an EKG for patients older than 40 years (American Psychiatric Association, 2002). Within the first 6 months of treatment renal function should be assessed 2–3 times, and thyroid function should be assessed once or twice. For the remainder of the treatment period, renal function and thyroid function should be tested 1–2 times a year or as clinically indicated (American Psychiatric Association, 2002).

Unlike the antiepileptic agents, lithium is not known to impact enzymes involved in medication metabolism. Therefore, it is not known to have pharmacokinetic interactions with atypical antipsychotics or anti-epileptics. There may be a risk of increased additive side effects when combining lithium with other psychotropics, however, the majority of the interactions that either increase or decrease the lithium level occur with non-psychiatric medications. Lithium is a salt and is renally excreted, so medications that impact free water or electrolyte balances, as well as those that impact renal excretion, may impact lithium. Notably, lithium levels can be increased and lead to toxicity if lithium is combined with nonsteroidal anti-inflammatory medications (Scherf-Clavel et al., 2020). As these are commonly taken over-the-counter medications, patients should be advised about this interaction. Lithium may also be impacted by a number of cardiovascular medications, including some used to treat high blood pressure and heart failure. There have been data to suggest that thiazides, loop diuretics, angiotensin-converting enzyme inhibitors, and angiotensin-receptor blockers may all lead to increased levels of lithium (Scherf-Clavel et al., 2020). As the therapeutic window for lithium is narrow, it is important to be aware of what other medications a patient is being prescribed prior to starting lithium as well as changes to their medication regimen, in order to avoid accidental toxicity.

### Fluoxetine

Fluoxetine, a selective serotonin reuptake inhibitor (SSRI), is commonly used to treat depressive and anxiety disorders and has been shown to be effective in treating PIA. Its ease of administration, favorable side effect profile, and efficacy in treating impulsivity in IED make it an ideal first choice for Type 1 aggression. While it has a more tolerable side effect profile than some of the mood stabilizers or antiepileptics, it is not free of side effects.

The class of SSRIs is generally thought to have a similar side effect profile. However, a meta-analysis reviewing side effects from fluoxetine compared to other SSRIs and tricyclic antidepressants (TCAs) showed that fluoxetine was better tolerated when compared to TCAs, but not in comparison to other SSRIs (Brambilla et al., 2005). The side effects caused by SSRIs are thought to be primarily due to increased serotonin throughout the body as there are serotonin receptors in multiple different organ systems. Fluoxetine was noted to cause insomnia, agitation, tremor, and anxiety more frequently than other antidepressants (Brambilla et al., 2005). Common side effects in other organ systems include gastrointestinal upset, sweating, dizziness, and weight change, primarily weight loss (Brambilla et al., 2005). While fluoxetine is noted to be activating, it may cause higher levels of sedation at higher doses, which may be of interest when considering prescribing practices for PIA (Rosenbaum and Ionescu, 2017). While some of the side effects from fluoxetine are transient, one of the prolonged and possibly underestimated side effects is sexual dysfunction. A meta-analysis review of studies of treatment-emergent sexual dysfunction with antidepressants, including fluoxetine, suggested rates of overall sexual dysfunction as high as 70% (Serretti and Chiesa, 2009). The reviewers acknowledge that there were a limited number of studies, particularly with fluoxetine, that may be impacting rates.

Over the last few decades, there has been some conflicting information regarding the teratogenic potential of fluoxetine. In 1997, Goldstein et al. (1997) prospectively identified pregnancies with first-trimester fluoxetine exposure and compared outcomes to historical reports of newborn surveys. They found that there was no consistent pattern of malformations observed to suggest teratogenicity, additionally, they noted major malformations in 3.5% of pregnancies that were consistent with the expected 4.0% rate in the general population. In 2015, Reefhuis et al. (2015) analyzed a dataset from the National Birth Defects Prevention Study and found associations between ventricular septal defects, right ventricular outflow tract obstruction cardiac defects, and craniosynostosis. The reviewers note, however, that these results need to be corroborated with an independent data source, and if the associations are found to be causal, the overall increase in absolute risk remains low. In 2016, a meta-analysis investigated the safety of fluoxetine during pregnancy and found that when women were using fluoxetine in the first trimester, there were a statistically significant increase in major malformations, particularly cardiovascular malformations. This analysis noted an increase in cardiovascular defects with a relative risk of 1.36, septal defects with a relative risk of 1.38, and non-septal defects with a relative risk of 1.39 (Gao et al., 2016).

One of the benefits of utilizing fluoxetine as a first-line agent in the treatment of PIA, particularly Type 1, is its ease of administration. Fluoxetine administration does not require baseline laboratory work to be done, nor does it require periodic monitoring of blood work. It is administered once a day, and its long half-life makes it unlikely that people will experience withdrawal symptoms if they stop the medication abruptly.

The majority of fluoxetine’s drug–drug interactions are thought to occur secondary to its role as an enzyme inhibitor. It is known to be a potent inhibitor of CYP2D6 and therefore may lead to increased levels of co-administered medications that rely on this enzyme for their metabolism (Rosenbaum and Ionescu, 2017). There are many medications metabolized by CYP2D6, including many tricyclic antidepressants, beta-blockers, haloperidol, sertraline and thioridazine, all of which may have increased levels if co-administered with an inhibitor like fluoxetine (Manzi and Shannon, 2005).

## Cost and Convenience of Each

Within the criminal justice system in which mental health services are subject to limited support and the offender population is largely indigent, and without health insurance, the cost of medication can affect its availability. If a medication is available and prescribed both during custody and after release into the community, low cost favors continuity of treatment. Accordingly, the cost of each AIAA is a consequential consideration.

The cost of medications, in general, will vary based on a patient’s insurance and the manufacturer. The following information, which reflects average retail prices, was based on UpToDate, which utilizes the pricing information gathered by the Medi-Span database, as well as information from GoodRx, which is a free-to-use company that tracks prescription drug prices in the US and offers various discounts and coupons.

The pricing of prescription medication will change greatly based on different formulations and whether the generic or a brand name form is being prescribed. The focus here is on the generic and most commonly prescribed formulations of the medications. All of the agents identified as effective in the treatment of PIA are relatively old and affordable. Of the agents discussed above, lithium seems to be the cheapest (possibly as low as $10 for a month’s supply), and oxcarbazepine may be the most expensive (possibly around $100 for a month’s supply). Those prices can be brought even lower with coupons available from websites like GoodRx if a patient will be paying out-of-pocket (GoodRx, 2021; UpToDate, 2021). If a patient does have insurance, some formulation of these medications will likely be covered, making the cost even lower. Cost can and should be considered on an individual basis, however, in general, the agents that have been shown to be effective in treating PIA are not cost-prohibitive.

As discussed above, there are no FDA-approved agents for the treatment of PIA. While we have focused on evidence supporting AIAA, there are many other agents commonly prescribed for impulsive aggression, although studies to support their efficacy in treating PIA are lacking. Notably, atypical antipsychotics are frequently prescribed in both correctional and civil settings for impulsive aggression. While the evidence-supported AIAAs are relatively affordable, the atypical antipsychotics often used come at a higher cost. Olanzapine and risperidone may start at $160 and $60 a month, and newer atypical antipsychotics such as aripiprazole may cost up to $650 a month for patients paying out-of-pocket. Coupons may be available for atypical antipsychotics as well, but on average, prices are higher for these newer medications (GoodRx, 2021).

## Discussion

From this review, our most remarkable finding is that there have been no further drug trials on the five AIAAs that met the quality parameters of the earlier review. Therefore, after 8 years, phenytoin, carbamazepine/oxcarbazepine, valproate/divalproex, lithium, and fluoxetine remain as the most evidence-based agents for the treatment of PIA. Nonetheless, further investigation on these agents is needed if they are to be advanced towards FDA approval for the treatment of IED/PIA. Longer-term drug trials are needed to determine whether these agents can lose efficacy after extended use, as has been observed by certain psychotropic and antiepileptic agents in the treatment of conditions for which they have been FDA approved.

Future drug trials could examine other agents which are already in use for treatment of impulsive aggression like other SSRIs such as sertraline (Feder, 1999), escitalopram (Cremers et al., 2016), citalopram (Towbin et al., 2020), and other antiepileptic mood stabilizers such as lamotrigine (Labiner et al., 2009). Taking into account their risk–benefit ratios, antipsychotics such as risperidone (Chengappa et al., 2000; Aleman and Kahn, 2001; Moeller and Swann, 2007), olanzapine (Bozzatello et al., 2017), and clozapine (Buckley et al., 1995; Rabinowitz et al., 1996; Glazer and Dickson, 1998; Krakowski et al., 2006; Moeller and Swann, 2007; Mela and Depiang, 2016) can be studied following the drug trial protocol for AIAAs. Clozapine has been shown to have a strong anti-aggression effect, not only in schizophrenia spectrum disorders but also in men with psychopathy (Brown et al., 2016), but the serious and sometimes uncomfortable side effects can be a disadvantage in achieving optimal safety and compliance.

One class of agents, called “serenics,” gained interest in the 1980s and 1990s for their potential anti-aggressive effects. These agents, of the phenylpiperazine class, include eltoprazine, fluprazine, and batoprazine and act as 5-HT1A and 5-HT1B agonists (Van der Poel et al., 1982; Olivier et al., 1990; Schipper et al., 1990), a mechanism of action which has been implicated in their anti-aggressive properties (Sijbesma et al., 1991). Initial animal models, primarily on rodents, showed promising anti-aggressive action on several different subtypes of aggression, including maternal defensive aggression, isolation-induced intruder aggression, target biting behavior, and father-to-offspring aggression (Benton et al., 1983; Flannelly et al., 1985; Olivier et al., 1985; Parmigiani and Palanza, 1991; Mos et al., 1992). These agents were also shown to be safe and well-tolerated (Van Harten et al., 1990). Further research revealed anxiogenic effects like that of stimulants (Griebel et al., 1990; Rodgers et al., 1992), as well as a shift to the right in aggression measures and dose-related REM sleep suppression following prolonged exposure (Carelli and Wagner, 1988; Quattrochi et al., 1993). In humans, no clinical trials meeting our inclusion criteria were conducted. The Eltoprazine Aggression Research Group in 1994 found no clinical effectiveness in a 28-week the study of 160 mentally handicapped participants with different psychiatric conditions (De Koning et al., 1994), although it did not differentiate impulsive aggression from other types of aggression.

From this review, novel agents have been tested for efficacy in treating not PIA but related conditions such as anger, hostility, and irritability. Such agents include 5-aminolevulinic acid phosphatase (Higashikawa et al., 2020), fish oil (Dean et al., 2014), and specific omega-3-fatty acids (Bellino et al., 2013), the 5-HT_2c_ agonist lorcaserin (Coccaro and Lee, 2019), the antiepileptic tiagabine (Gowin et al., 2012), the benzodiazepine clobazam tested in Lennox–Gastaut Syndrome (Paolicchi et al., 2015), and the glutamate NMDA receptor antagonist memantine (Kulkarni et al., 2018). As an antagonist for neurokinin-1 (NK1) receptors upon which substance P acts, aprepitant, used as an antiemetic, could theoretically function as an AIAA but has yet to be tested (Fanning et al., 2021).

A critical question, for which we only have speculation, is why there have been no quality studies of AIAAs since 2013 and so few since Sheard’s classic lithium study over four and one-half decades ago (Sheard et al., 1976). One factor, observed before and again in this review, is the common methodologic flaw in studies that the aggression studied is not defined and diagnosed as impulsive or IED (Felthous and Barratt, 2003). Authorities have long recommended using an agent known to be effective in the treatment of another disorder for which the person may have some symptoms. Not necessarily invalid, but if this is the only guideline to go by, clinicians and investigators may not separately diagnose PIA. The culture of prison correctional and service employees supports traditional disciplinary measures to manage aggressive measures and may have no interest in drugs, although the AIAAs are not agents that are commonly misused. Finally, consider delusional disorder, a serious mental illness that can have behavioral and forensic consequences. In the landmark case on involuntary pharmacotherapy for restoration of competence to stand trial (Sell v. U.S., 2003), the American Psychiatric Association supported the use of antipsychotic medication (American Psychiatric Association, 2003), whereas the American Psychological Association rejected this approach (American Psychological Association, 2002) in their respective briefs to the Supreme Court. In contrast to the pharmacotherapy of impulsive aggression, we are aware of no cross-over double-blind studies having been conducted to determine the efficacy of any agent for the treatment of this disorder (Felthous et al., 2001; Smith and Buckley, 2006; Skelton et al., 2015). For whatever reason, some disorders are simply not sufficiently investigated neurobiologically and pharmacologically.

## Conclusion and Future Direction

Five drugs remain as having shown evidence for efficacy in the treatment of PIA: phenytoin, carbamazepine/oxcarbazepine, valproate (Depakote/Divalproex), lithium, and fluoxetine. Yet more research is needed on each of these before any are advanced to FDA approval for PIA or IED, and other candidate drugs remain to be adequately tested for efficacy in treating PIA. In selecting an agent for the treatment of PIA with an AIAA, current information is needed on the severity of outbursts, material risks, and side effects, efficacy and vulnerability to adverse effects in special populations, and cost and convenience of administration of each agent, all of which have been summarized here.

Finally, there is reason to believe that impulsive aggression may be a risk factor for recidivism and failure of released prisoners to successfully reintegrate into the community, yet there is a striking dearth of data on the prevalence of PIA and its importance as a risk factor for released criminal offenders, including those whose index offense could have been impulsive in nature. Although some research supports assessment and pharmacotherapy to reduce institutional violence resulting from untreated PIA, research on conditional release and parole outcomes is virtually devoid of this inquiry, creating investigative opportunities for potential improvement in criminal rehabilitation.

## Author Contributions

AF, BM, JN, RD, EK, FC, and MS took part in the process of creating this manuscript, satisfying the criteria provided by the editorial standards in relation to autorship. Specifically, BM and JN gave a more substantial contribute in the writing process. AF was involved in the conception of the manuscript and gave substantial contributes during the reviewing process, other than being the corresponding author. RD, EK, and MS were dedicated to the research and collection of data from previous research and in the final approvation of the manuscript. Finally, FC was involved in the writing process and in the editing of the manuscript following the editorial rules. All authors contributed to the article and approved the submitted version.

## Conflict of Interest

The authors declare that the research was conducted in the absence of any commercial or financial relationships that could be construed as a potential conflict of interest.

## Publisher’s Note

All claims expressed in this article are solely those of the authors and do not necessarily represent those of their affiliated organizations, or those of the publisher, the editors and the reviewers. Any product that may be evaluated in this article, or claim that may be made by its manufacturer, is not guaranteed or endorsed by the publisher.
